# Case report: Paraneoplastic lower motor neuronopathy associated with a malignant liver tumor

**DOI:** 10.3389/fneur.2024.1325318

**Published:** 2024-02-12

**Authors:** Chaowei Xu, Hanfan Wu, Jian Chen

**Affiliations:** ^1^Department of Neurology, Affiliated Jinhua Hospital, Zhejiang University School of Medicine, Jinhua, China; ^2^Department of Electro Neurophysiology, Affiliated Jinhua Hospital, Zhejiang University School of Medicine, Jinhua, China

**Keywords:** motor neuron disorders, paraneoplastic lower motor neuronopathies, malignant tumor of the liver, diagnosis, treatment

## Abstract

Paraneoplastic lower motor neuronopathies (LMNs) have rarely been reported with malignant liver tumors. A 71-year-old man developed chronic progressive upper limb and cranial nerve paralysis. Electromyography examination suggests chronic progressive neuronal damage involving the right C4–T1 nerve root innervated muscle and the right sternocleidomastoid muscle. Magnetic resonance imaging suggested the presence of a malignant liver tumor. His serum was positive for anti-Yo antibodies. Hepatic artery chemoembolization was performed, followed by treatment with pembrolizumab and lenvatinib. The patient’s condition improved, and paraneoplastic LMNs were diagnosed. Paraneoplastic causes should be considered in the differential diagnosis of chronic progressive LMNs. A combination of surgical treatment and immunotherapy may result in a favorable outcome.

## Introduction

Motor neuron disease (MND) is a fatal neurodegenerative condition that is characterized by the selective loss of the upper and lower motor neurons (1). First of all, we need to rule out other diseases that cause the same symptoms before we can make the diagnosis, such as paraneoplastic neurological disorders. Paraneoplastic neurologic syndromes (PNSs) are associated with cancer and are characterized by an immune-mediated pathogenesis supported by specific neuronal antibodies (2, 3). Paraneoplastic motor neuron disorders have been reported in the past; the condition may improve after cancer treatment (4). Cases of paraneoplastic LMNs caused by liver malignant tumors have rarely been reported in the literature. This study aimed to demonstrate that liver malignant tumors can cause LMNs and represent potentially treatable conditions.

## Case report

The patient is a 71-year-old man with a history of chronic hepatitis B and cirrhosis. He was admitted to our center with a 6-month history of right upper limb paralysis and bulbar paralysis. The patient’s limb movements and swallowing function deteriorated slowly. There was no significant weakness in his left upper limb or both lower limbs. His pronunciation was unclear, and he had no pharyngeal reflexes bilaterally. There were no sensory symptoms or sphincter problems. The weakened muscles were mainly located on the right side of the body, including the sternocleidomastoid, trapezius, deltoid, biceps, brachioradialis, radial wrist flexor, extensor digitorum, extensor digitorum propria, abductor pollicis brevis, and dorsal interosseous muscle. The muscle strength was 4/5 on the MRC scale, and the other three limbs were normal. Fasciculations were detected in the right upper limb and occasionally in the lower limbs. Sensory examinations, including pain, temperature, vibration, and position sense, were normal. Romberg’s test was negative. He did not have a Babinski sign or a sign of an exaggerated deep tendon reflex.

**Figure 1:**
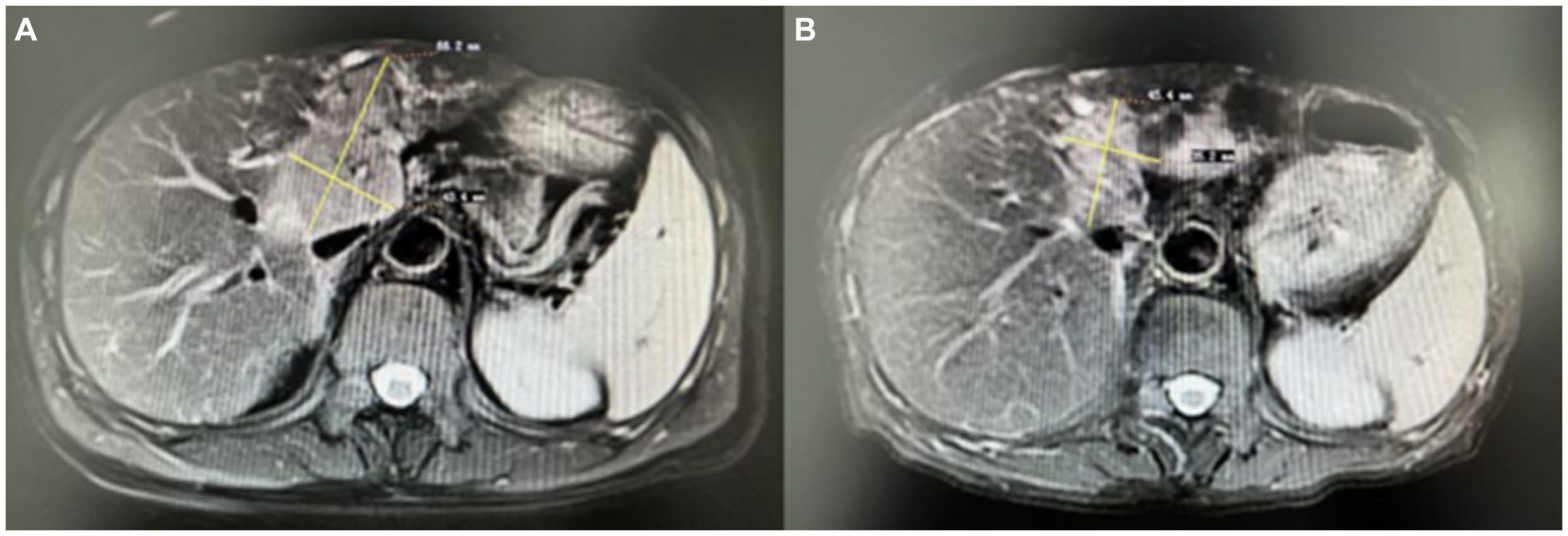
Liver MRI with gadolinium contrast showed the presence of a malignant tumor before treatment **(A)**, and after treatment, the size of the liver tumor was reduced **(B)**.

A nerve conduction study showed low amplitudes for median and ulnar nerve CMAPs in the right upper limbs, whereas SNAPs were normal. There was no conduction block. Needle electrode examination demonstrated fibrillation potentials and positive sharp waves, and the amplitude of MUP increased, involving the right C4-T1 nerve root innervated muscle and the right sternocleidomastoid muscle. These findings suggested chronic progressive neuronal damage. Liver MRI with gadolinium contrast showed the presence of a malignant tumor (Figure 1A). Cervical MRI showed slight protrusion of intervertebral disks on 5/6 and 6/7, with no significant compression damage to the nerve roots or spinal cord. Cerebrospinal fluid (CSF) analysis showed normal cell count and protein concentrations. No oligonucleotide bands were detected in either CSF or serum. The CSF cytology was negative for neoplastic cells. Serum testing for erythrocyte sedimentation rate, rheumatoid factor, O antigen, nuclear antigen antibodies, anti-ganglioside antibodies, anti-SSA antibodies, anti-Ro-52 antibodies, vasculitis antibodies, immunofixation, and protein electrophoresis were normal. Serum infection indicators, including AIDS, Epstein–Barr virus, cytomegalovirus, *Toxoplasma gondii*, syphilis, and Lyme disease, were negative. A panel of neuronal autoantibodies revealed the presence of anti-Yo antibodies (Hangzhou Oumengwei Medical Laboratory). There was no evidence of upper motor neuron damage; therefore, the paraneoplastic LMNs were considered.

Hepatic artery chemoembolization was performed, followed by treatment with pembrolizumab and lenvatinib. Pembrolizumab was injected every 3 weeks, and oral lenvatinib was initiated at the same time, once a day. The patient’s condition improved, and the liver tumor size was reduced (Figure 1B). The results of the electromyography examination were slightly worse; the nerve conduction velocity and the amplitude of CMAPs decreased; however, the distribution of nerve damage did not expand. The patient has been followed up on for 2 years. On the most recent visit, the muscle strength in both upper limbs was 5/5 on the MRC scale. The Kubota water swallowing test grade was improved.

## Discussion

We report a case of chronic progressive motor neuronopathy preceding the diagnosis of a malignant liver tumor. Our case yields two significant observations. First, chronic progressive motor neuronopathies may occur in patients with malignant liver tumors. Second, surgical treatment combined with immunotherapy may improve the associated neurological disability. On the contrary, there is currently no effective treatment for neurodegenerative diseases.

Paraneoplastic neurologic syndromes are characterized by nervous system inflammation, indolent tumor growth, and an immune reaction against antigens shared by the nervous system and the tumor cells (5–7). Diagnosing paraneoplastic LMNs may be difficult, as the incidence of paraneoplastic LMNs is low and many other causes need to be ruled out. It is easy to miss a diagnosis in clinic work. However, correctly identifying patients with paraneoplastic LMNs is crucial, as this represents a potentially treatable condition. A French series identified eight cases of probable paraneoplastic MND out of 2,200 consecutive cases diagnosed with MND over 5 years, following cancer treatment and immunoglobulin, 5 (62.5%) were in remission (4).

The etiology of motor neuron disorders includes infectious, neoplastic, inflammatory, paraneoplastic, genetic variation, toxic, and degenerative causes (8, 9). This report demonstrates that liver malignant tumors can cause motor neuron disorders. The electrophysiologic features, serum, and CSF studies, and improved neurological function after effective treatment for malignant tumors helped establish the diagnosis in our patient. After 2 years of follow-up, the patient’s condition was improved by the treatment of the tumor, which confirmed that this is not a degenerative process. In addition, we ruled out tumor metastasis, infectious disease, inflammation, and toxic exposure through medical history inquiry and examination. After a long follow-up, there was no evidence of upper motor neuron damage. The diagnosis of paraneoplastic LMNs was made.

The primary features of paraneoplastic MND can be summarized as follows (4): (1) subacute nature; (2) lower motor neuron syndrome, associated or not with upper motor neuron involvement; (3) predominant asymmetric upper limb involvement; (4) presence of other non-motor neurological manifestations, including sensory neuronopathy; (5) signs of inflammation in the CSF; and (6) neurological improvement or stabilization after immunotherapy and tumor treatment. Our patient presented with asymmetric lower motor nerve damage in the upper limb, a characteristic presentation. CSF inflammation is common in PNS patients and can be helpful for diagnosis, especially if this analysis is performed within 3 months after neurological onset (10). Our patient’s CSF was normal, possibly due to the chronic course of the disease. His condition improved after surgical treatment and immunotherapy, which helped us make a correct diagnosis.

Anti-Yo antibodies are closely associated with paraneoplastic cerebellar degeneration (PCD) in the setting of gynecological and breast malignancies, and the antibodies cause Purkinje cell death by binding to the intracellular Yo antigen (11). A case of esophageal adenocarcinoma with PCD was reported, and anti-Yo antibodies were detected in the serum (12). Paraneoplastic ALS were reported in a female with ovarian cancer, and anti-Yo antibodies were also detected in the serum (13). In this study, we report a very rare case of anti-Yo antibodies associated with paraneoplastic LMNs arising in patients with liver malignant tumors.

Diagnosing paraneoplastic LMNs may be difficult because of the low incidence, and we should rule out many other causes. However, identifying these syndromes is critical, as treatment can manage these potentially fatal diseases.

## Data availability statement

The datasets presented in this article are not readily available because of ethical and privacy restrictions. Requests to access the datasets should be directed to the corresponding author.

## Ethics statement

The studies involving humans were approved by the Ethics Committee of Jinhua Central Hospital. The studies were conducted in accordance with the local legislation and institutional requirements. The participants provided their written informed consent to participate in this study. Written informed consent was obtained from the individual(s) for the publication of any potentially identifiable images or data included in this article.

## Author contributions

CX: Investigation, Writing – original draft. HW: Data curation, Investigation, Writing – original draft. JC: Conceptualization, Resources, Supervision, Writing – review & editing.
